# Human Rabies: A Reemerging Disease in Costa Rica?

**DOI:** 10.3201/eid0906.020632

**Published:** 2003-06

**Authors:** Xiomara Badilla, Victor Pérez-Herra, Ligia Quirós, Ana Morice, Edwin Jiménez, Elizabeth Sáenz, Fernando Salazar, Rodrigo Fernández, Lillian Orciari, Pamela Yager, Sylvia Whitfield, Charles E. Rupprecht

**Affiliations:** * Caja Costarricense de Seguro Social (CCSS), San José, Costa Rica; † Hospital Nacional de Niños, San José, Costa Rica; ‡ Ministerio de Agricultura y Ganadería, San José, Costa Rica;; § Instituto Costarricense de Investigación y Enseñanza en Nutrición y Salud (INCIENSA), San Isidro de Pérez Zeledón**,** Costa Rica; ¶ Epidemiología, Región Brunca, CCSS, Tres Ríos, Cartago**,** Costa Rica; # Epidemiología, Región Brunca, Ministerio de Salud de Costa Rica, San Isidro de Pérez Zeledón; ** Centers for Disease Control and Prevention, Atlanta, Georgia, USA

**Keywords:** human rabies, bat, Costa Rica, dispatch

## Abstract

Two human rabies cases caused by a bat-associated virus variant were identified in September 2001 in Costa Rica, after a 31-year absence of the disease in persons. Both patients lived in a rural area where cattle had a high risk for bat bites, but neither person had a definitive history of being bitten by a rabid animal. Characterization of the rabies viruses from the patients showed that the reservoir was the hematophagous Vampire Bat, *Desmodus rotundus*, and that a sick cat was the vector.

Multiple studies in North and South America (*1*–*3*), Africa (*4*), Australia (*5*), Asia (*6*), and Europe (*7*) have documented the importance of rabies maintenance by various bat species. Aerosol transmission from bats was once considered as a possible mechanism in human rabies acquisition; however, because most patients lack a documented history of an animal bite, a more plausible explanation is that people, unaware of the risk of acquiring rabies from bat bites, are bitten by infected bats (*8*–*10*). The last human rabies case in Costa Rica, before the case we document, occurred in 1970, before the control of rabies in domestic animals had improved. The last documented case of canine rabies occurred in the North Pacific Coast (Salinas Bay) in 1987, and 26 cases of cattle rabies caused by Vampire Bat bites were reported during the 1990s. In September 2001, after 31 years without any known case of human rabies in Costa Rica, the National Children’s Hospital reported a suspected case of paralytic rabies in a child referred from a regional hospital in the Brunca Region.

## Case Report

On September 12, 2001, a 9-year-old boy from La Gamba, Río Claro, in the Brunca Region of Costa Rica, was admitted to a local hospital with fever, cough, and malaise of 4 days’ duration. On September 19, he returned to the hospital because of muscular weakness; he and was referred to the regional hospital on September 23 with acute flaccid paralysis that rapidly progressed to respiratory failure. The patient was intubated and transferred to the National Children´s Hospital with a diagnosis of viral encephalomyelitis. His severe central nervous system involvement progressed to coma in <24 h with muscle hypotonia. A computed tomographic scan showed generalized brain edema, and an electroencephalogram indicated diffuse and severe cerebral damage. The child died 6 days after hospital admission on September 29. A nuchal skin biopsy tested negative for rabies virus antigen by an immunofluorescence antibody assay, but postmortem brain tissue tested positive. Rabies virus was isolated by injecting mice with brain tissue from the child.

**Figure 1:**
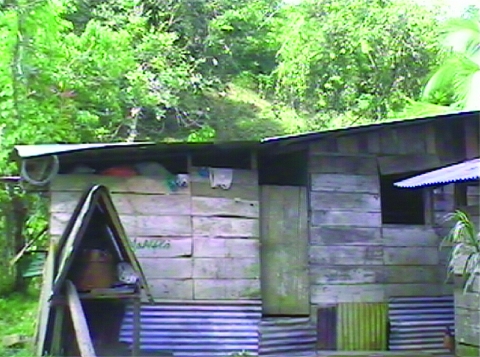
Patient’s home, located in an area where the risk of being bitten by a bat was high.

A field investigation was carried out to identify the likely mechanism of transmission. The house where the child had lived during the previous 4 months was in a wooded zone (Figure 1). In an interview, two members of the family noted that a 62-year-old woman who was in charge of the child died in a similar manner. On September 17, she exhibited malaise, muscular pain, headache, insomnia, and anxiety. On September 23, she was admitted to the local hospital, but because of the severity of her disease, she was transferred to the regional hospital on September 26. On September 29, she had sialorrhea, hemiplegia, and loss of muscular strength in her right arm; she also showed bizarre behavior and respiratory failure that progressed to apnea. She died on September 30. Characterization of the agents, conducted at the Centers for Disease Control and Prevention, demonstrated a rabies virus variant associated with vampire bats (*Desmodus* *rotundus*) from both patients, as described elsewhere in Latin America (*11*).

After the diagnosis of rabies was made, additional history was obtained about potential animal exposures. The family reported that during the middle of July 2001, their cat became very aggressive and demonstrated strange behavior. Their dog attacked the cat, and while the woman and child tried to stop the fight, the cat bit them. The dog killed the cat, and the carcass was discarded into a creek next to the house. (The body of the cat was not found during the investigation.) After the human cases were reported, the dog was confined with close observation, and in December 2001, it was euthanized. Postmortem brain tissue samples from the dog tested negative for rabies virus antigen.

**Figure 2:**
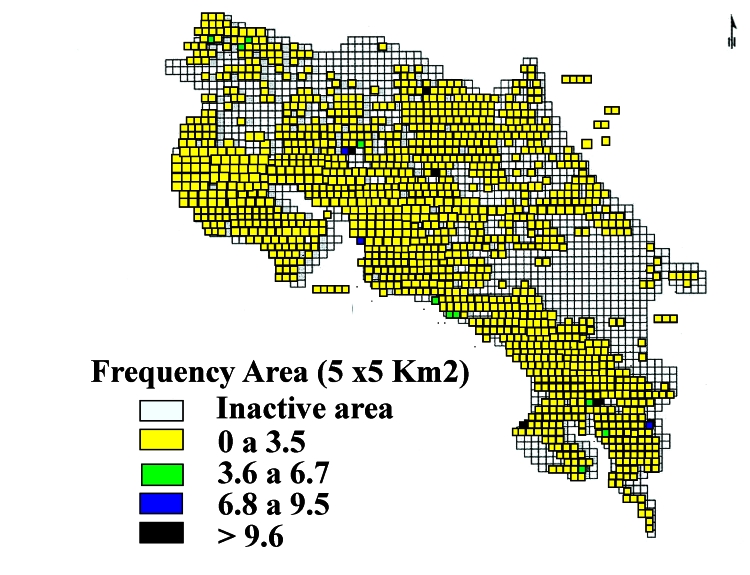
Annual prevalence of Vampire Bat bites, Costa Rica, 2001.

**Figure 3:**
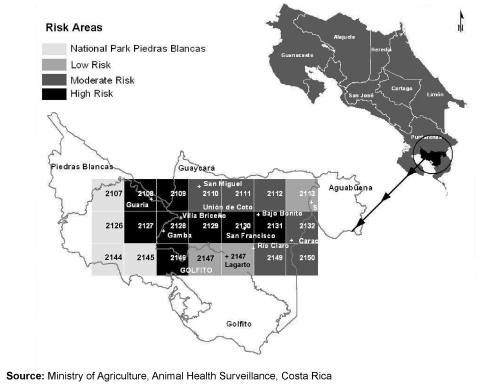
Human rabies cases, Costa Rica, September 2001.

Because rabies occurs in cattle in Costa Rica and because persons living in areas where contact with Vampire Bats is likely are at risk for of viral transmission, the Program of Animal Health of the Ministerio de Agricultura y Ganadería had implemented a Geographic Information System (GIS) during 1980. This animal surveillance system divided the country into 2,234 small areas (5 x 5 km^2^). Trained veterinary technicians used a national census of animals to register the number of weekly Vampire Bat bites from the farms included in each area. According to the frequency of Vampire Bat bites in cattle, the presence of rabies virus in populations of bats, and ecologic variables (e.g., temperature, precipitation, altitude, and land use), the GIS program defines areas of low, medium, and high-risk of disease (Figure 2). During August and September 2001, the GIS of animal health surveillance reported 169 cattle bites by Vampire Bats in the Brunca Region (rate 3.5%). Where the two patients lived, the rate of such attacks was 10.4%, and the area was classified as a high-risk zone for bat bites to cattle (Figure 3).

Rabies postexposure prophylaxis with a Vero cell rabies vaccine was administered to the relatives of the patients and to veterinary and healthcare personnel, who were in contact with the saliva of the patients. A retrospective search of other possible human cases was performed by analyzing the deaths with unknown origin in the discharge registry of the hospitals of the Brunca Region and the registry of the National Morgue. The case definition used was the following: any acute encephalopathy of unknown origin, with atypical focal neurologic signs or encephalomyelitis in any person who died between January 1 and September 30, 2001, in the Brunca Region. The search did not find any additional suspicious deaths associated with rabies in the region.

## Conclusions

After 31 years without a case of human rabies in Costa Rica, the disease reemerged. Its reappearance reinforces the importance of an integrated network between veterinary and human health programs to identify risk zones of bat bites and to prevent human and animal cases. The active participation of combined health services in the early warning of the occurrence of suspected cases of emergent diseases is essential for effective, integrated surveillance. Human rabies must always be considered in the differential diagnosis of acute flaccid paralysis with consciousness impairment, even in patients without a known history of animal bite (giving priority to those in zones where cattle are at high risk for bat bites).

Rabies in bats was first reported during the 1920s. Since then, rabies has been confirmed in several species of bats. The historical association between Vampire Bats and livestock may tend to subvert the epidemiologic surveillance in other species, such as domestic animals such as cats and dogs (*12*). This lack of association with other species could be due to a lack of intensified surveillance or to unknown factors related to the virus, host, or ecology.

Several studies have documented the transmission of rabies virus in the absence of a history of documented animal bite (*13*,*14*). No typical bite marks were observed in either of the patients, but the history of cat bite raises the possibility of an indirect manner of transmission, through the bite of another vector, previously infected by a bat. At the community level, the immigration of human populations throughout rural areas increases the risk of contact with hematophagous bats and facilitates the transmission of the virus to humans.

The presence of rabies in humans in Costa Rica associated with bat bites has demonstrated the importance of strengthening the integrated surveillance of animal and human health care. The GIS operation permits the identification of risk zones, so that measures for optimal control and prevention can be established. Participation of communities located in high-risk areas is a key strategy for the prevention and early detection of possible cases of rabies. Emergence of human rabies from infected wildlife, especially in countries where the disease has been eliminated in dogs, reinforces the need for maintaining awareness of both physicians and public health workers alike, as well as for improving laboratory tools needed for rapid rabies diagnosis.
